# A Deterministic Model for Determining Degree of Friendship Based on Mutual Likings and Recommendations on OTT Platforms

**DOI:** 10.1155/2022/9576468

**Published:** 2022-06-30

**Authors:** Aqeel Khalique, Mohammad Khalid Imam Rahmani, Mohd Saquib, Imran Hussain, Abdul Wahab Muzaffar, Mohd. Abdul Ahad, Md Tabrez Nafis, Mohd Wazih Ahmad

**Affiliations:** ^1^Department of Computer Science & Engineering, Jamia Hamdard, New Delhi, India; ^2^College of Computing and Informatics, Saudi Electronic University, Riyadh, Saudi Arabia; ^3^ASTU, Adama, Ethiopia

## Abstract

In recent years, the application of various recommendation algorithms on over-the-top (OTT) platforms such as Amazon Prime and Netflix has been explored, but the existing recommendation systems are less effective because either they fail to take an advantage of exploiting the inherent user relationship or they are not capable of precisely defining the user relationship. On such platforms, users generally express their preferences for movies and TV shows and also give ratings to them. For a recommendation system to be effective, it is important to establish an accurate and precise relationship between the users. Hence, there is a scope of research for effective recommendation systems that can define a relationship between users and then use the relationship to enhance the user experiences. In this research article, we have presented a hybrid recommendation system that determines the degree of friendship among the viewers based on mutual liking and recommendations on OTT platforms. The proposed enhanced model is an effective recommendation model for determining the degree of friendship among viewers with improved user experience.

## 1. Introduction 

**Figure 1:**
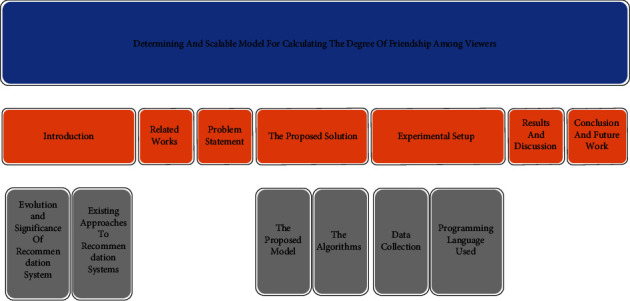
Overall structure of the study.

During the last couple of years, Amazon Prime, Netflix, Disney + Hotstar, and several other OTT platforms have emerged. Viewers have changed their watching patterns. The majority of the viewers rely on recommendations or ratings of movies. These watching patterns have been widely exploited by OTT platforms to gain a significant rise in popularity among their customer base. Viewers are privileged to use recommendations that are made available in the recommendation system adapted by the OTT platforms. The trend of recommender systems has increased due to an avalanche of various Internet services such as e-commerce to suggest buyers some interesting commodities that they might find useful to buy and online promotion to suggest the users some content that might be matching their preferences. Nowadays, recommender systems are almost unavoidable during our online activities. Naturally, users feel more confident to find recommendations accurate and reliable when they come from a known or likable person. Recommender systems have the flexibility to forecast whether users will prefer items based on their personal preferences and choices. A recommendation system is like a data shortlisting process to prioritize the items based on users' preferences discovered from their use patterns. A recommendation system is an algorithm that aims to recommend related items to users, where the items can be movies to watch, texts to read, products to buy, or any other content that depends on the application field. The recommendation systems have much potential to increase revenue for business organizations dealing in almost every area of application. So, their important features need to be harnessed effectively [1]. The organization of the study is depicted as shown in Figure 1.

**Figure 2:**
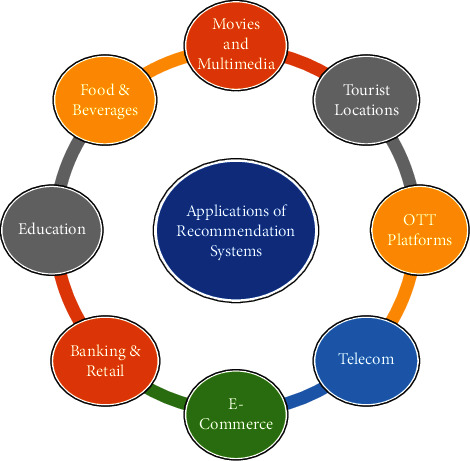
Applications of recommendation systems.

There exist numerous recommender systems covering widespread usage and application areas.  Figure 2 shows different application areas of recommendation systems.

### 1.1. Evolution and Significance of Recommendation System

The humongous growth in the accessibility of digital content and an exponential increase in Internet usage have brought a massive data overload due to which timely access to accurate information is delayed. Information retrieval giants such as Google and Bing have nearly resolved this problem, but still lack prioritization and personalization of data (the system maps available content to users' interests and preferences).

**Figure 3:**
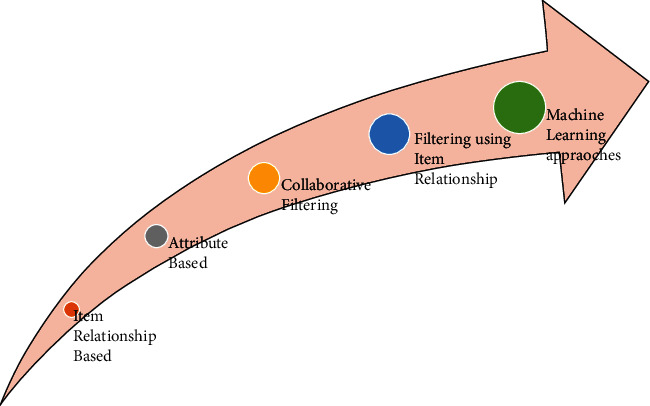
Evolution of recommendation systems based on different techniques.

This increases the demand for recommender systems over the years [2]. Recommender systems have evolved from item relationship-based to machine learning approaches, and hence, their prediction has been significantly improved.  Figure 3 shows the evolutionary techniques of recommender systems as used over the past few years.

Recommendation systems' personalization features improve user involvement and retention. These systems capture users' experience and enhance their recommendations whether it can be product recommendations on an e-commerce site, movie, or music recommendations on streaming services such as OTT platforms [3]. The major reason behind the popularity and growing state of recommendation systems is that the users have multiple options available to choose from. Due to the exponential growth of social networking services, people tend to utilize these available services online. Similarly, on OTT platforms, users have plenty of choices, and hence, it is difficult for any user to decide what recommendation to follow [4–6].

### 1.2. Existing Approaches to Recommendation Systems

Recommendation systems need to know the personal preference of the user for effective suggestions. Information on user interactions is used to create a recommendation system. Therefore, data collection from different activities namely direct interactions and indirect interactions is very important. Direct interactions include information about the user's previous activities, ratings, comments, and information about the user's profile, such as gender, age, or interests. Indirect interactions include the user's device, clicked links, location, and date [7]. The working principle of the recommendation system is to find patterns in the user's previous practice. User characteristics, such as demographic data such as age and gender, and psychological characteristics, such as interests and dislikes, can help identify customers. Product features such as movie type and actors can help calculate the similarity between products [8]. There are three basic methods used to develop recommendation systems namely content-based, collaborative filtering, and hybrid methods. For example, in Netflix, instead of browsing through thousands of packages and movie titles, Netflix provides the user with a specific item as per his likeness. This feature can save time and provide a better user experience further reducing the cancellation rate and hence saving the company about a billion dollar annually [9].

#### 1.2.1. Collaborative Filtering

**Figure 4:**
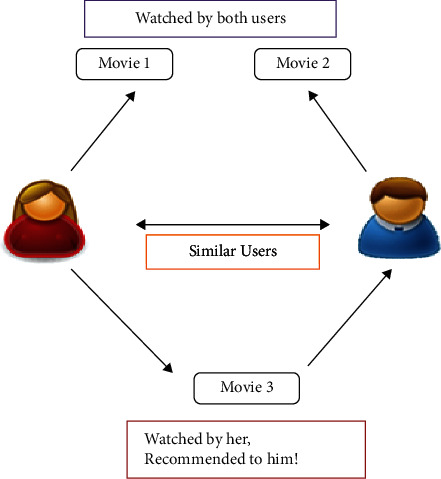
Collaborative filtering.

It is based on gathering and inspecting data on users' actions, habits, or likeness and forecasting based on their similarity with other users. Collaborative filtering does not depend on the content of the data, and it recommends without understanding the content of the item. As shown in Figure 4, collaborative filtering is based on historical likings of items resulting in similar types of items in the future.

#### 1.2.2. Content-Based Filtering

It is based on the details of the item and the interaction and preferences of individual users. Recommendations are based on the user's history and interactions. It uses algorithms to recommend items that are similar to the user's earlier liking.

**Figure 5:**
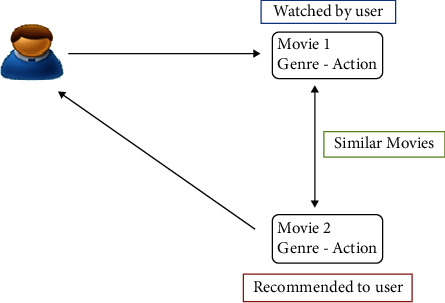
Content-based filtering.

Accuracy depends on the available information about the data. In some industries due to privacy and regulatory issues, users' data and transactional data are not available sometimes resulting in a cold start problem. As shown in Figure 5, in content-based filtering, if a user likes an item, he will also like a “similar” item.

#### 1.2.3. Hybrid Recommendation Systems

**Figure 6:**
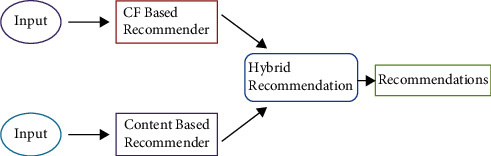
Hybrid recommendation systems.

The hybrid recommendation systems are an amalgamation of a collaborative filtering approach and a content-based filtering approach. In Netflix, hybrid recommender systems are used by comparing the viewing and search habits of similar users and providing movies that share characteristics with highly rated similar movies. Cold start and data scarcity problems are resolved in the hybrid technique.  Figure 6 shows the model of hybrid recommendation systems.

### 1.3. Phases of Recommendation Systems

**Figure 7:**
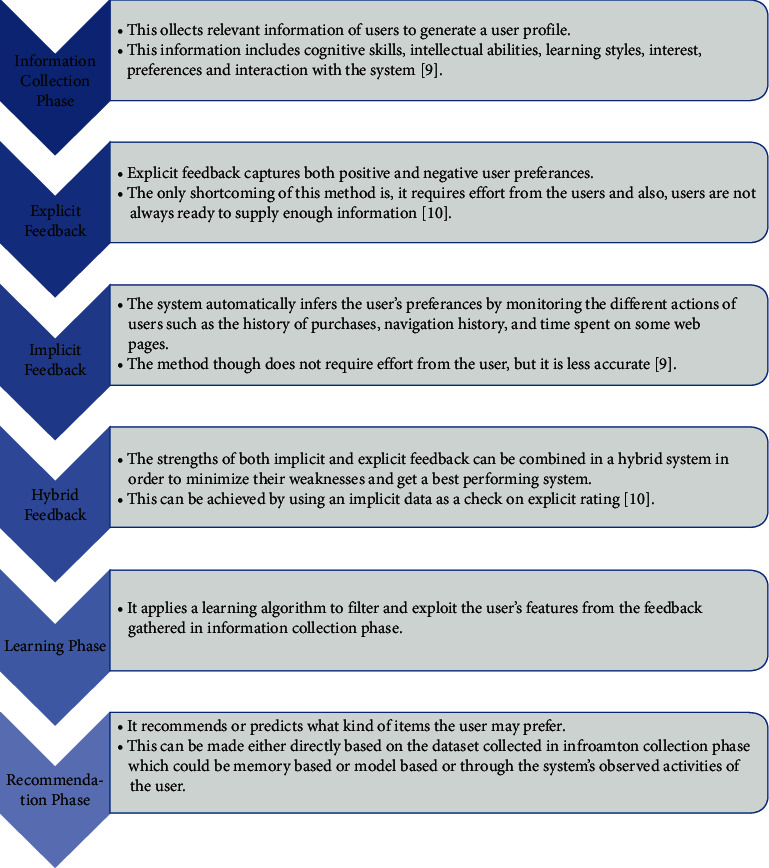
Phases of recommendation.

Recommendation systems can be implemented in several phases. These phases are shown in Figure 7.

## 2. Related Works

This section presents current state-of-the-art technology of recommendation systems with social connections between users. Trust-aware recommendation systems are more popular because social trust provides user preferences along with ratings of items. With the advent of social networking sites, personal information in social networks is used to predict user behavior. These methods assume that there are social networks between users and recommend users based on ratings provided by users who are connected with other users. Several research papers were studied and analyzed, and they have been summarized as shown in Table 1.

**Figure 8:**
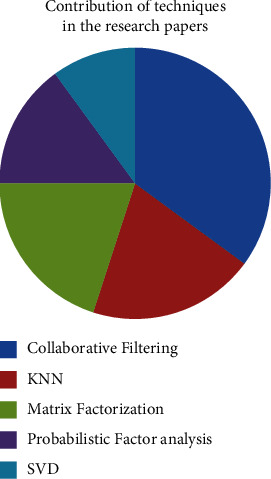
Contribution of recommendation techniques in related papers.

The contribution of these techniques to recommendation systems is shown in Figure 8. However, as we can observe, the majority of the recommendation systems were based on collaborative filtering. There is a need of the hour to determine recommendations that shall be scalable and more resilient in terms of social interactions, social bonding, or likings. These social attributes are much required when recommendations are required in OTT platforms.

## 3. Problem Statement

Nowadays, recommendations are mostly used in every aspect. Recommending users with the most suitable product is important to enhance user satisfaction. Generally, on OTT platforms, users do not know the importance of mutual liking and its impact on their degree of friendship. So, it is very important to establish an effective relationship between users of recommendation systems to exploit the advantages of their mutual likings for better user experience and satisfaction. For instance, if a user “a” watches a movie on an OTT platform and assumes that three of his other friends also watch movies on the same OTT platform, then it is important to determine the similarity of recommendations among these three users to the user “a”; besides, it will be helpful to determine the mutual likings among the three users. The similarity of likings is not available on OTT platforms for users. However, based on this mutual liking, a set of recommendations can be improved because, generally, people accept the recommendations of persons who have more similar likings in common. Hence, a deterministic and scalable model is much needed to offer more precise and correct recommendations to the users. Furthermore, based on the similarities of mutual likings, there will be more precise and correct recommendations. Therefore, there is a need to determine the degree of mutual friendship among the users. This study proposes a novel and unique model to address these problems.

## 4. The Proposed Solution

### 4.1. The Proposed Model

As discussed in the problem statement, we have proposed a deterministic model for computing the degree of friendship among viewers based on mutual liking and recommendations. Our work is an extension of [25]. In this research work, we have implemented an elaborated model for OTT platforms. However, the system can be implemented for every type of recommendation system. We have focused on the social relationships among connected users over any social networking services and further determine the mutual liking and degree of friendship among these socially connected users. We have formulated the objectives below and aimed our system to gather results based on these objectives during the implementation of the research work.

**Figure 9:**
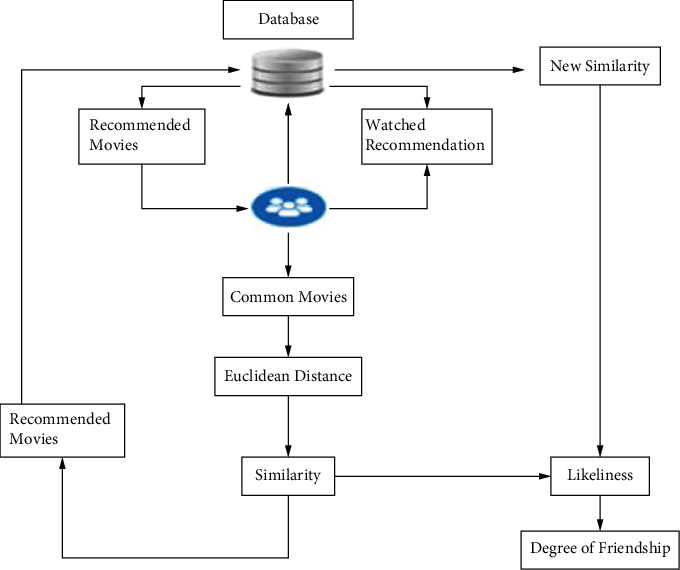
Proposed model.

- Objective 1 [O_1_]: similarity identification between user “a” and user “b” from the movie ratings given by them
- Objective 2 [O_2_]: recommendation of movies to user “a” from other users socially connected to the user “a”
- Objective 3 [O_3_]: determining mutual liking between two users
- Objective 4 [O_4_]: determining the degree of friendship between two users
- Based on our objectives, we have presented our work in [25] and further extended the work to achieve objectives 3 and 4.
- The proposed model is represented diagrammatically in Figure 9.
- Based on the objectives defined in an earlier section, we have designed the algorithms to achieve these objectives. Taking an exemplary scenario, the proposed model is explained below in detail.
- Firstly, we have user details in our system that have watched some movies and give them ratings after watching, and the system will find the common movies that they all have watched before.
- Secondly, the system will determine the Euclidean distance using the ratings of movies that the users gave to commonly watched movies. Using the Euclidean distance, the system will calculate the similarity between users. The reason behind using the Euclidean distance metric for determining the similarity identification between two users is because it is simple to implement and can give the difference in ratings given by different users for commonly watched movies. Other mechanisms for calculating similarity are cosine similarity, Pearson's correlation coefficient, K-means clustering, the Jaccard method, etc.
- Thirdly, the system will recommend movies to the concerned user; for instance, if the user “a” has the highest similarity with user “b” then the system will recommend movies from the watching history of user “b.”
- Fourthly, user “a” watches the recommended movies and gives them ratings, resulting in a slight change in the similarity between users. There are two types of similarities defined in our proposed model, *S*_1_ is the similarity among users before watching the recommended movies and *S*_2_ is the similarity among users after watching the recommended movies. The difference between the two similarities *S*_1_ and *S*_2_ is defined as mutual liking.
- Lastly, the degree of friendship is the product of mutual liking and an intersection between recommended movie sets of two concerned users.

Our proposed recommendation system uses a modular approach, as we have devised several algorithms and used these algorithms to fulfill the research objective mentioned earlier. Firstly, we determine the similarity between users regarding common movies in Algorithm Obj_1_, fulfilling O_1_. Secondly, in Algorithm Obj_2_, we recommend movies to the specific user based on the similarity. After the recommendations, the user might have finished watching the recommended movies and usually did the rating of the watched movies also. Hence, we again determine a new similarity among those sets of users using Algorithm Obj_1_, fulfilling O_2_. Thirdly, to determine the mutual liking we use Algorithm Obj_3_ by subtracting old similarity from new similarity, fulfilling O_3_. Fourthly, we use Algorithm Obj_4_ to determine the degree of friendship by multiplying the value of mutual liking with many commonly recommended movies between users, fulfilling O_4_. The section below presents the algorithms used in the proposed system.

### 4.2. The Algorithms

In our model, Algorithm Obj_1_, fulfilling O_1_, and Algorithm Obj_2_, fulfilling O_2_, were presented in [25]. As shown in algorithms 1 and 2, we present Algorithm Obj_3_, fulfilling O_3_, and Algorithm Obj_4_, fulfilling O_4._

Global Assumptions: we have assumed the following three sets:

 
*U* = {*u*_1_,…, *u*_*n*_}, the set of users
 
*R* = {*r*_1_,…, *r*_*n*_}, the set of ratings
 
*M* = {*m*_1_,…, *m*_*n*_}, the set of movies

## 5. Experimental Setup

We have implemented our model with Python on the Google Colab platform and a cloud-based Jupyter Notebook environment.

### 5.1. Data Collection

Data collection is a very important task for our implementation. We created our dataset by questionnaire method asking for the users' ratings through Google forms. Those users were connected through the social network also. Our dataset of 4000 records has a username, a list of movies watched, and ratings (1 to 5) for every watched movie given by the user. We cleaned it and used it for our research. From the dataset, we have created a data dictionary. The data dictionary has a user name, a list of movies watched, and ratings for every watched movie.

### 5.2. Programming Language Used

We used Python programming language to implement our approach. Python focuses on the core functions of the application by handling functional tasks. In Python, it is easier to maintain the code and the application [27].

## 6. Results and Discussion

**Figure 10:**
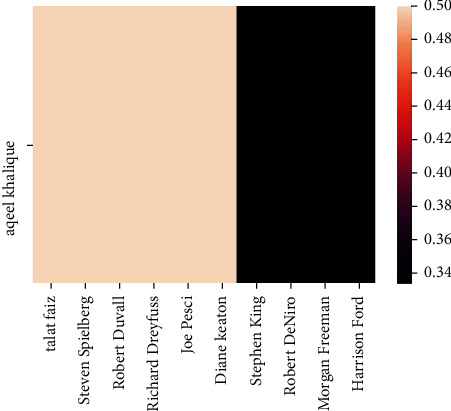
Similarity, *S*_1_ between the specified user and the rest of the users before watching the recommended movie.

**Figure 11:**
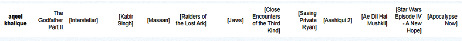
Recommendation suggested by the proposed model for the specified user based on similarity.

**Figure 12:**
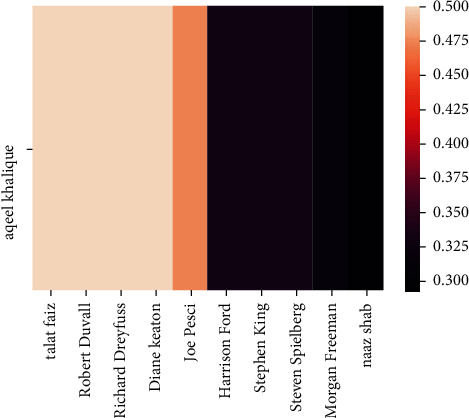
Similarity *S*_2_ among users after they have finished watching the recommended movies.

The implementation shows that we have achieved our objectives successfully, and as the mutual liking among the recommendations will increase, the degree of friendship between the users will also enhance. Therefore, for an instance and instinctively, if there is a greater degree of friendship among two users, it will be highly likely to follow the recommendations suggested by the latter to the former. Also, the friendship between two users can be determined based on their mutual liking. These results are presented below from the executing environment of Google Colab.  Figure 10 shows the similarity S1 of user “aqeelkhalique” with other users in the database before user “aqeelkhalique” watched any recommended movie. Figures 11 and 12 show the recommendation of movies and similarities after the user has finished watching the recommended movies.

**Figure 13:**
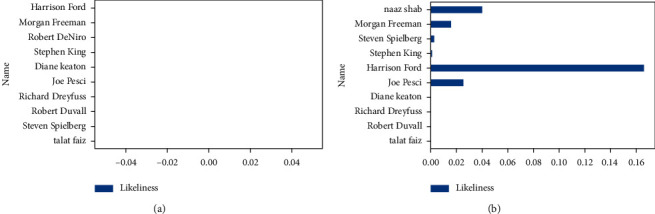
(a) Mutual likings between the specific users and the rest of the users before the user started watching the recommended movies. (b) Mutual likings between the specific user “aqeelkhalique” and the rest of the users after the user “aqeelkhalique” finished watching the recommended movies.

Figures 13(a) and 13(b) show mutual liking among user “aqeelkhalique” and the rest of the users before watching recommended movies and submitting ratings after watching recommended movies, respectively.

**Figure 14:**
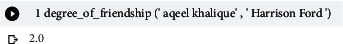
Degree of friendship.

Figure 14 finally shows the degree of friendship between “aqeelkhalique and Harrison Ford” after determining mutual liking among the concerned users.

The degree of friendship as determined in the results clearly indicates the relationship between mutual liking and similar recommendations. There exist several other mechanisms for recommending movies. However, determining the degree of friendship makes our proposed methodology unique. Hence, based on mutual liking, an effective recommendation system can determine the degree of friendship or social bond among users. Also, once mutual liking will increase, subsequent recommendations can be more effective as it depends on mutual liking and the personal preferences of users. The model can be scalable with the growing dataset as the number of users and ratings for watched movies will increase substantially. Prominent giants such as Netflix also used cold start and survey/questionnaires initially to generate recommendations [28, 29].

## 7. Conclusion and Future Work

In OTT platforms, it has been observed that the mutual liking between users can enhance the recommendation system's performance. In this study, we have proposed a hybrid approach to adapt the factors to user preferences and social connections to make accurate recommendations. Generally, when a user watches a movie suggested by the system, the mutual liking of the users for the movies may vary accordingly. Using mutual likings, we have defined a degree of friendship between users. With the help of our proposed recommendation system, the user will be able to know that among his/her friends who are closer or likable to him/her determined by the degree of friendship. Our model is novel and unique in its algorithmic approach and has never been implemented on any OTT platforms so far.

The results indicate that mutual likings and degree of friendship information are beneficial in recommending movies on an OTT platform. Our proposed model is novel and has been implemented using our algorithms. The experiments performed have a limitation on the available dataset as the proposed method will require scalable information about realistic user ratings.

Future work may not be limited to including intelligent insights based on analytics, optimization methods, and scalable real-time dataset to integrate different types of information and other factors to improve the performance of the recommendation systems. The influence of social networking may result in more accurate recommendations due to similar interests, tastes, mutual likings, etc.

## Figures and Tables

**Algorithm 1:**
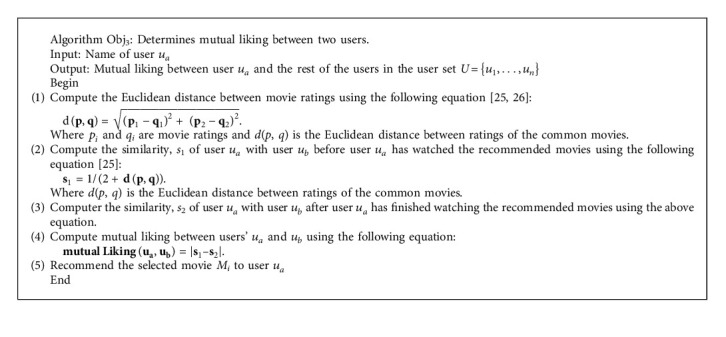
Algorithm Obj_3_.

**Algorithm 2:**
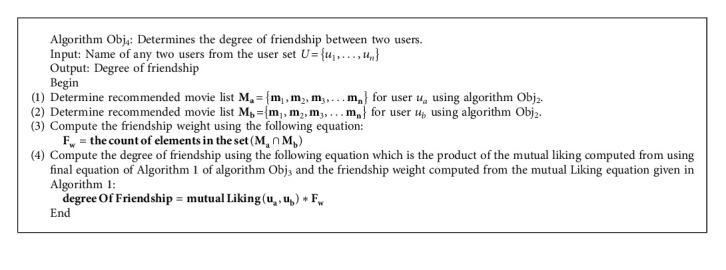
Algorithm Obj_4_.

**Table 1:** Related work.

S. no.	Paper reference	Techniques used	Key findings	Observations
1	[10]	Probabilistic factor analysis	Determines authenticity and the realistic nature of news	Calculates the probability of news from news data sets collected by different websites
2	[11]	Collaborative filtering	Describes profile similarity and trust-based recommendation systems	A correlation between trust and the largest single difference in score exists along with overall similarity
3	[12]	Collaborative filtering	Describes social networks based on semantic networks and trust for movie recommendations	Creates predictive rating recommendations for movies
4	[13]	KNN	Includes multigraph ranking model	Results represent different user relationships in multiple graphs and recommend the nearest neighbors of specific users
5	[14]	Probabilistic factor analysis	Includes trust-based recommendations in a social network	It has lower recommendation probabilities
6	[15]	KNN	Includes a hybrid approach	Combines user ratings and social trust. Compared with other trust-aware recommendation work, their method uses untrusted links and investigates their dissemination effect
7	[16]	Collaborative filtering	Includes user relevance and evolutionary clustering	Correlation is calculated by combining user satisfaction and potential score information
8	[17]	Collaborative filtering	Includes a new similarity calculation method called JacRA	Calculates selection of the items and the ratings. It has complex calculations to determine ratings
9	[18]	Collaborative filtering	It solves data scarcities, cold start, recommendation accuracy, and timelines as an improved collaborative recommendation algorithm	It involves the traditional similarity of the collaborative recommendation algorithm, for an improved recommendation
10	[19]	Matrix factorization	Includes a hybrid approach combining social behavior, movie genre, and existing collaborative filtering algorithms	Calculates similarity of movies to predict user ratings
11	[20]	Collaborative filtering	Includes collaborative filtering based on the ratings of the movies implemented by Apache mahout	Considers user ratings to recommend movies
12	[21]	Matrix factorization	Includes model-based approach using matrix factorization techniques in social networks	It resolves the cold start problem to some extent using social MF
13	[22]	Probabilistic factor analysis	Includes probabilistic factor analysis method that calculates multifaceted trust relationships and user profiles by sharing the user's potential feature space	It cannot generate better results using trust relations for predictions
14	[23]	Content-based, collaborative filtering	It includes a collaborative filtering approach and uses the information provided by users	It provides suggestions to the users using the two renowned algorithms
15	[24]	Matrix factorization	Includes dual role preferences (trustee/trustee specific preferences), and trust-aware recommendations are achieved by modeling explicit interactions	Using explicit interactions makes it difficult to compute due to privacy issues

## Data Availability

The data are available on request from the corresponding author.
